# The Cooking and Pneumonia Study (CAPS) in Malawi: Implementation of Remote Source Data Verification

**DOI:** 10.1371/journal.pone.0155966

**Published:** 2016-06-29

**Authors:** William Weston, James Smedley, Andrew Bennett, Kevin Mortimer

**Affiliations:** Liverpool School of Tropical Medicine (LSTM), Liverpool, Merseyside, United Kingdom; Glasgow University, UNITED KINGDOM

## Abstract

**Background:**

Source data verification (SDV) is a data monitoring procedure which compares the original records with the Case Report Form (CRF). Traditionally, on-site SDV relies on monitors making multiples visits to study sites requiring extensive resources. The Cooking And Pneumonia Study (CAPS) is a 24- month village-level cluster randomized controlled trial assessing the effectiveness of an advanced cook-stove intervention in preventing pneumonia in children under five in rural Malawi (www.capstudy.org). CAPS used smartphones to capture digital images of the original records on an electronic CRF (eCRF). In the present study, descriptive statistics are used to report the experience of electronic data capture with remote SDV in a challenging research setting in rural Malawi.

**Methods:**

At three monthly intervals, fieldworkers, who were employed by CAPS, captured pneumonia data from the original records onto the eCRF. Fieldworkers also captured digital images of the original records. Once Internet connectivity was available, the data captured on the eCRF and the digital images of the original records were uploaded to a web-based SDV application. This enabled SDV to be conducted remotely from the UK. We conducted SDV of the pneumonia data (occurrence, severity, and clinical indicators) recorded in the eCRF with the data in the digital images of the original records.

**Result:**

664 episodes of pneumonia were recorded after 6 months of follow-up. Of these 664 episodes, 611 (92%) had a finding of pneumonia in the original records. All digital images of the original records were clear and legible.

**Conclusion:**

Electronic data capture using eCRFs on mobile technology is feasible in rural Malawi. Capturing digital images of the original records in the field allows remote SDV to be conducted efficiently and securely without requiring additional field visits. We recommend these approaches in similar settings, especially those with health endpoints.

## Introduction

### Background

Malawi is a landlocked country in Sub-Saharan Africa. Ranked the 18^th^ least developed country in the World, 85% of its population is rural [1]. Malawi is characterized by a heavy burden of disease in childhood, with an under-5 mortality rate of 71 per 1000 births [2]. Pneumonia is one of the most common causes of death and morbidity in childhood in Malawi. Approximately 300 per 1000 children under five are diagnosed with the condition annually [3–4]. The domestic burning of biomass fuels, such as animal waste, charcoal, agricultural residue, and wood, is a major risk factor for childhood pneumonia [5]. In Malawi, where 95% of households rely on biomass fuels for cooking, heating and lighting, indoor air pollution is likely to account for a substantial burden of this disease [6]. Advanced cook-stove technologies can improve combustion efficiency and reduce smoke emissions by as much as 90% [7]. The Global Alliance for Clean Cookstoves was launched in 2010 and aims to introduce clean, efficient stoves, and fuels into 100 million homes by 2020 [8]. It is unclear whether this approach will bring health benefits. The Cooking And Pneumonia Study (CAPS) is a cluster randomized controlled trial of an advanced cook-stove technology to prevent pneumonia in children under five years of age in Malawi. CAPS is funded by the Medical Research Council (MRC), the Wellcome Trust, and the Department for International Development (DFID). The CAPS protocol, which includes, the study design, study population, allocation of interventions, sample size, outcome assessment, safety monitoring, training of fieldworkers, and trial management, can be accessed on the trial website www.capstudy.org [9] [10].

Data monitoring in clinical trials is a process that aims to ensure that data are complete, accurate, and verifiable [11]. In clinical trials, data are captured from source documents (original records) and recorded on the Case Report Form (CRF). The data recorded on the CRFs must be consistent with the source documents. Source Data Verification (SDV) is a data monitoring procedure by which the data in the CRF is compared for consistency with the data contained in the source documents. The process of conducting SDV also provides an opportunity for study protocol and Good Clinical Practice (GCP) monitoring to be performed. Two previous studies that evaluated the effects of biomass smoke exposure reduction on health outcomes did not report specifically on SDV [12–13]. A literature review published in 2008, demonstrated the importance of SDV, finding an average of 976 errors per 10,000 data values (from source to database) [14].

Traditionally, SDV relies on monitors making multiple visits to study sites requiring extensive travel. Bresclauer et al. reported that SDV accounts for 46% of the time spent on-site by monitors and 40% of the costs for phase II or phase III trials [15]. In another phase III cardiovascular clinical trial, on-site monitoring accounted for 25 to 30% of costs [16].

The widespread availability of low-cost mobile technologies has led to its growing use as a method for electronic data capture. Walther et al. compared commonly available methods of electronic data capture (a netbook, Personal Digital Assistant (PDA), and a small laptop computer) with conventional paper-collection methods in rural West Africa [17]. They found electronic data capture methods offered a fast, convenient, and cost effective approach for the entry, management, and reporting of data.

CAPS adopted mobile technology to capture data on an electronic CRF (eCRF). This allowed data to be accessed remotely and securely from the United Kingdom (UK). This avoids the heavy financial, human, and time resources associated with traditional paper-collection methods for on-site SDV. In the present study, descriptive statistics are used to report the experience of electronic data capture with remote SDV in a challenging rural setting of Malawi.

## Methods

CAPS is a 24-month village level cluster randomized controlled trial. The trial has recruited over 4500 households representing over 10000 children across two study sites, Chikhwawa District in the South and Karonga District in the North. Participants have been randomized to receive an advanced cook-stove intervention (two Philips fan-assisted stoves with a solar charger per household) or continuation of traditional cooking methods. The primary outcome is the incidence of pneumonia in children under the age of five. Healthcare providers, blinded to trial arm, diagnosed pneumonia using the World Health Organization (WHO) Integrated Management of Childhood Illness (IMCI) pneumonia assessment protocol [18].

The CAPS site we report on here is represented by villages of the remote and rural Chikhwawa district of Southern Malawi, about 30 miles from the city of Blantyre. Chikhwawa is a poorly resourced area with limited infrastructure. Households were recruited from populations mostly living in deprived conditions. To ensure a minimum of six months’ data collection before the child’s fifth birthday, all children up to the age of four and a half years living in the trial catchment area were eligible to participate in CAPS. CAPS began collecting data on the 1st of December 2014. As of 15th of June 2015, 664 episodes of pneumonia had been recorded and included in the present study.

The members of the CAPS team, involved in SDV were, one of its co-principal investigators, the trial manager, the data managers, the project manager, 22 fieldworkers, and the SDV worker. Compliance with the protocol and Standard Operating Procedures (SOPs) by field staff was maximized through training events to include GCP training and periodic quality control audits.

### Ethical considerations

CAPS was approved by the College of Medicine Research Ethics Committee (COMREC) in Malawi and LSTM Research Ethics Committee (REC) in the UK. Informed and voluntary written consent was obtained from the next of kin, caretakers, or guardians on behalf of the minors/children enrolled in CAPS. This was documented through consent forms that each participant signed. The ethics committees/Institutional Review Boards (IRB) approved this consent procedure.

### Identification of patients

Health workers, including health surveillance assistants, nurses, and medical officers, employed in local facilities, such as health posts, village clinics, health centers, private health clinics, and district hospitals, were responsible for the clinical assessment, classification, and management of pneumonia. Patients were diagnosed according to the WHO IMCI pneumonia protocol.

**Fig 1:**
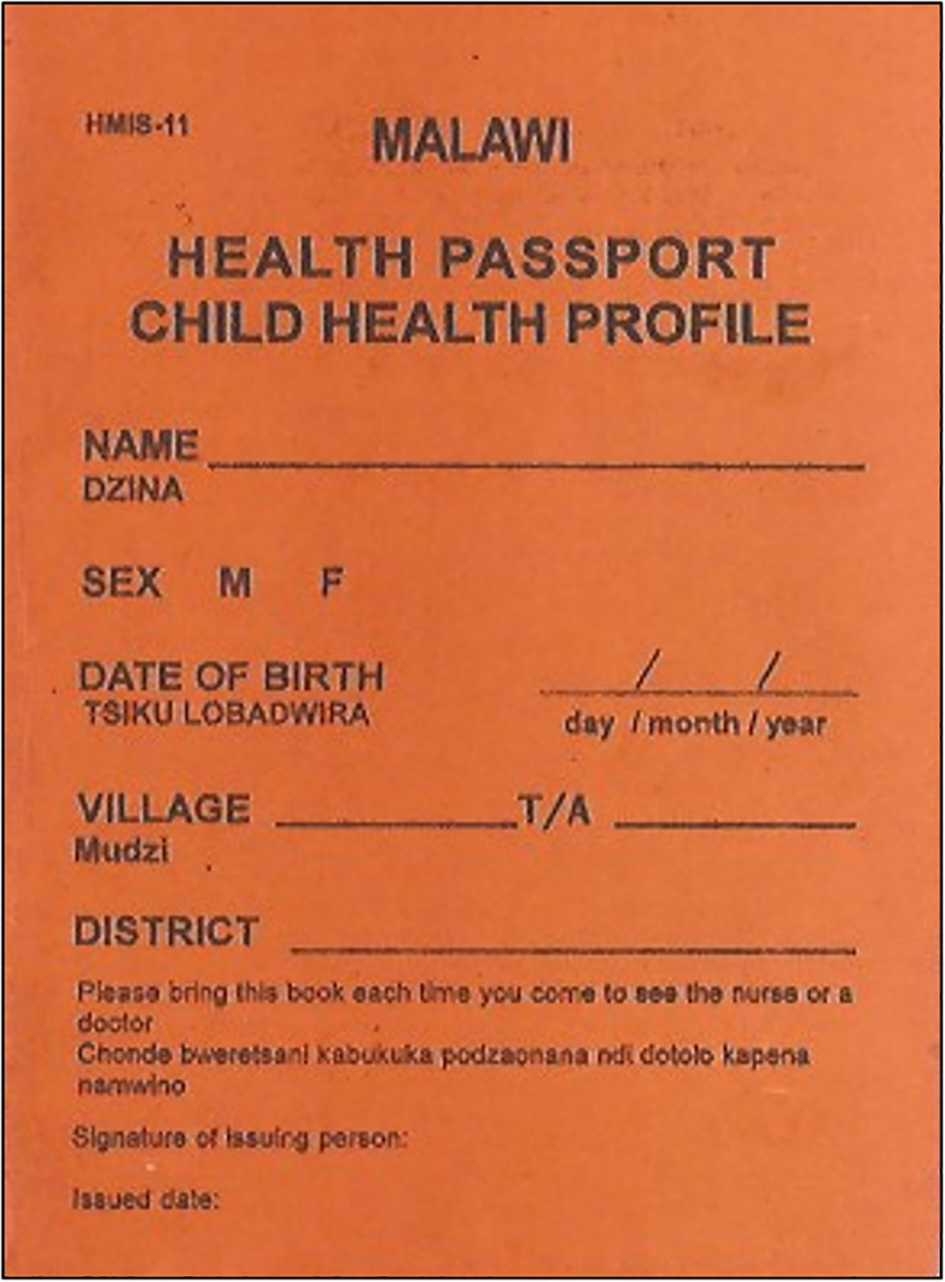
The cover page of the health passport.

**Fig 2:**
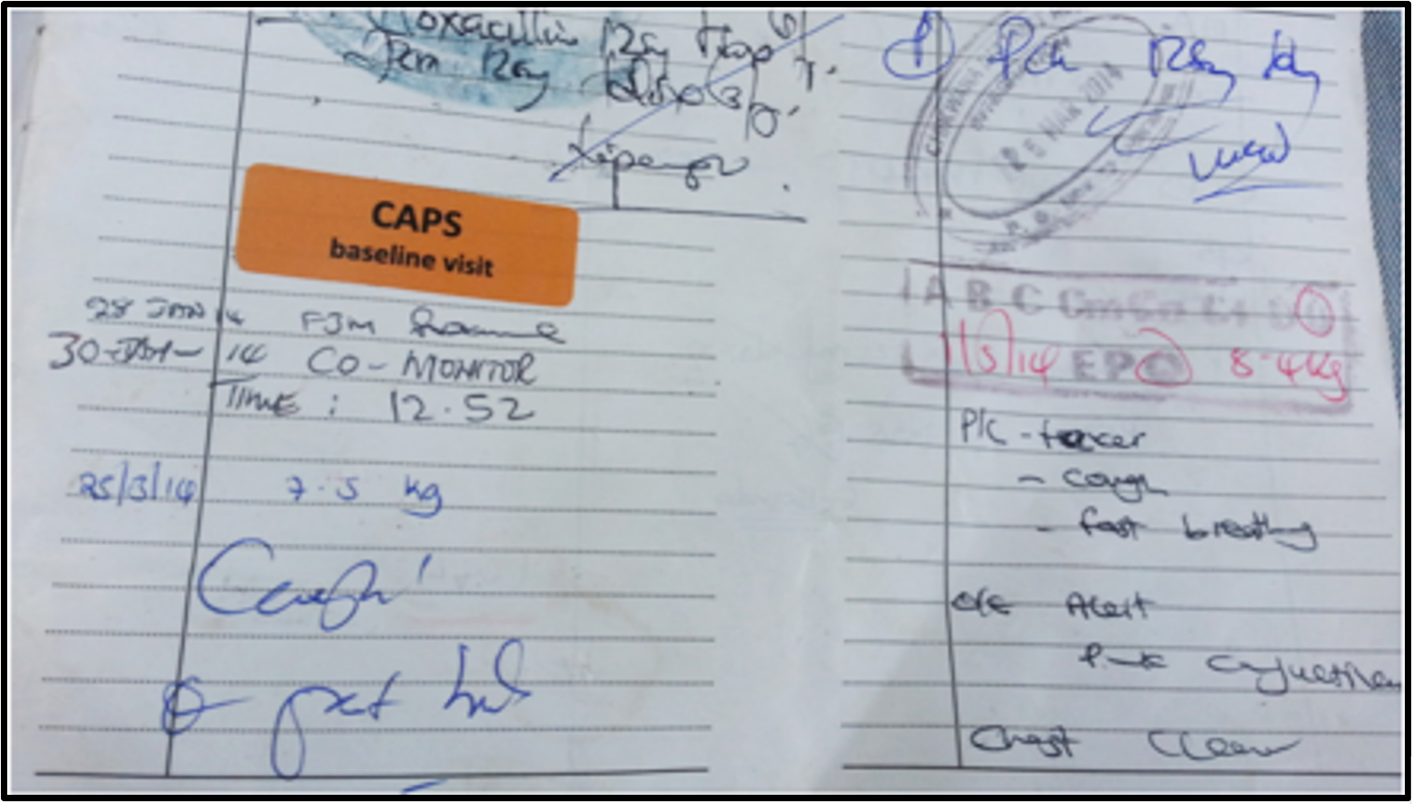
A record of a health related event in the health passport.

In Malawi, if any child presents to a health facility, health workers make a record of the event (including the assessment, classification, and management plan) in a free government booklet called the health passport (Fig 1) (Fig 2). CAPS facilitated high quality documentation by providing all participants with a new health passport, if they did not have one with a sufficient number of blank pages.

**Fig 3:**
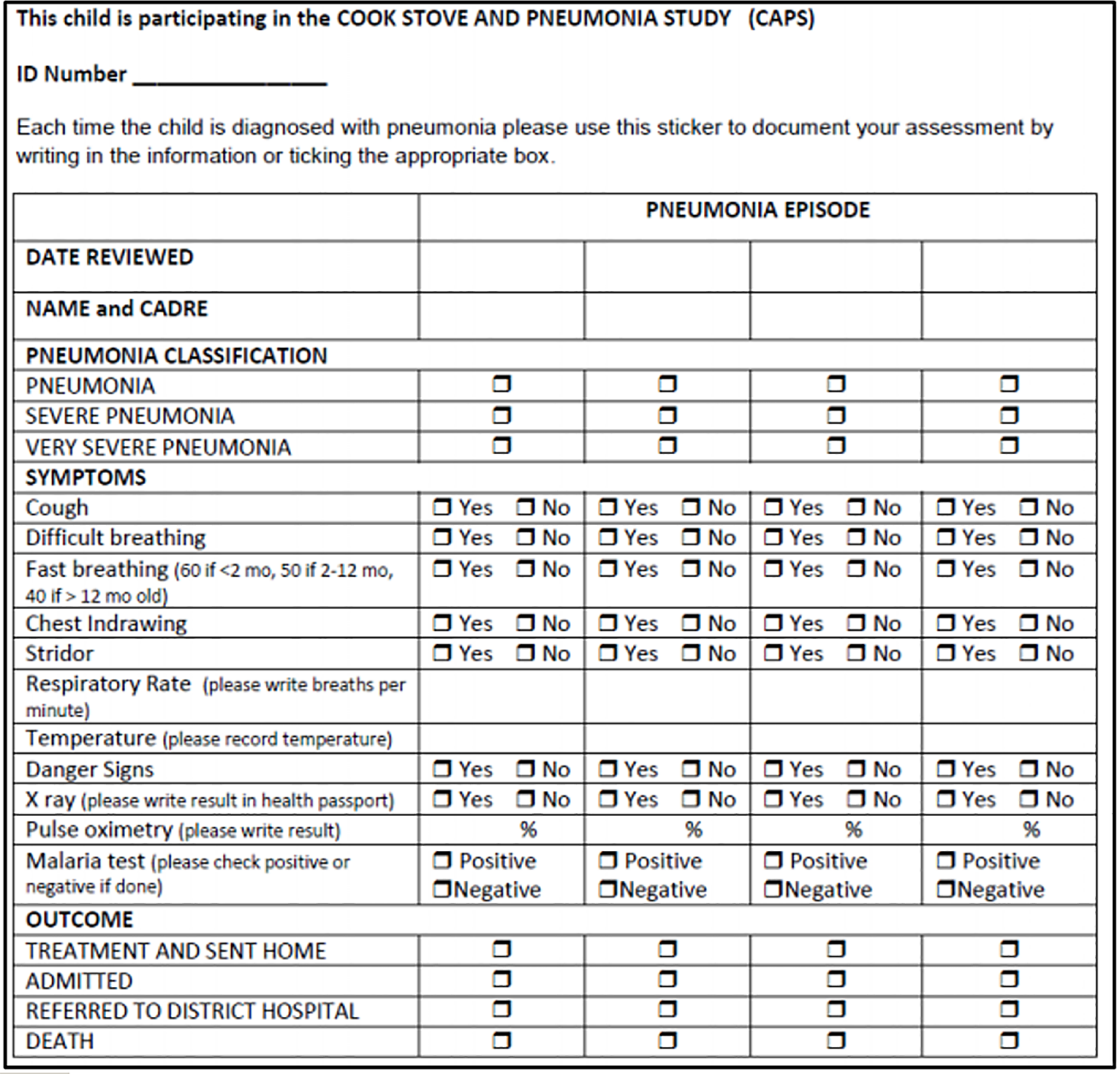
The study-specific CAPS sticker.

A study-specific CAPS sticker was inserted in the health passports of all participants. The CAPS sticker informed the health worker that the child was enrolled in CAPS. In addition to recording the event in the health passport, if an episode of pneumonia had occurred, health workers were asked to record the date, classification, clinical indicators (cough, temperature, respiratory rate, chest in-drawing, stridor, danger signs, difficulty breathing, and fast breathing), and outcome of the episode as outlined in the CAPS sticker (Fig 3).

#### Data capture

At three monthly intervals, fieldworkers employed by CAPS, visited participants at their homes to collect data from the health passport and CAPS sticker (source documents). Fieldworkers also captured digital images of the pages of the source documents relating to the last study visit, up to the latest entry. Data were collected using an eCRF on a Samsung Galaxy S3 Smartphone. Use of the eCRF allowed data to be entered directly from the study site into the master database, making the large number of CRFs manageable.

If an episode of pneumonia had been recorded in the health passport since the last study visit, the fieldworkers recorded its occurrence in the eCRF. If the same episode of pneumonia recorded in the health passport had been recorded in the CAPS sticker, fieldworkers transcribed the data from the CAPS sticker into the eCRF. If multiple episodes were recorded in the health passport and/or CAPS sticker within one time-period month, only the first episode of pneumonia was recorded in the eCRF as it could be considered as the same infection.

### Data handling

The Open Data Kit (ODK) Collect mobile application was installed on each of the mobile devices as a platform for the eCRF. An eCRF in ODK was in the format of a XML file, ODK Collect interpreted this XML file and displayed it as a questionnaire on the mobile device. When the eCRF was completed, the data were stored in a separate XML file, which was saved to the storage of the mobile device. When Internet connectivity (either via wi-fi or mobile data) became available, the mobile device sent the XML file to the ODK Aggregate server, which was the server software responsible for collating all of the data. When the send was completed and the mobile device received confirmation of this, the XML file containing data was deleted.

The Malawi Liverpool Wellcome (MLW) programme provided an administrative and infrastructural hub for CAPS. The ODK Aggregate server was hosted at MLW within their data centre. The base operating system, on which ODK Aggregate ran, was UBUNTU. The database software used to store the data was MySQL. The programming of the eCRF was completed by a data manager at MLW. He was also responsible for the installation, programming and maintenance of the ODK Aggregate server at MLW. Initially, data had to be manually transported in a portable hard drive from Malawi to the UK. Later on, our server at LSTM was able to connect to the ODK Aggregate server at MLW to take a copy of the CAPS data.

Initially, Microsoft Access was used to present the data from the eCRF recorded for each episode of pneumonia in the master database with the digital images of the health passport and CAPS sticker. Microsoft Excel was used to record the results of SDV. We later developed a web-based SDV application using a combination of PHP and HTML. PHP is a programming language used to interpret data from MySQL so it can be displayed in a web page. Rather than accessing the data on computers in the LSTM, this allowed SDV to be conducted from anywhere on a password protected web page. This secure remote access provided a user-friendly platform and enabled comparisons to be made easily and in a timely manner.

### Data security

UBUNTU, MYSQL, and ODK aggregate were password protected. Access was restricted to authorized users only. All the mobiles devices and computers used in CAPS were password protected. The mobile devices were returned to the health center in Chikhwawa at the end of each day of data collection, where they were stored overnight and recharged. The data centre at MLW, where the ODK Aggregate server was hosted, was at a secure location managed by their Information Technology (IT) services.

### Source Document Verification from the UK

SDV was conducted for data critical to trial outcomes for all recorded episodes of pneumonia in the master database for:

- The occurrence of pneumonia (recorded in the health passport)
- The severity classification (recorded in the CAPS sticker)
- 8 clinical indicators (recorded in the CAPS sticker)

Each episode of pneumonia recorded in the master database was recognized as an SDV case, and given a unique SDV case number. If there were no discrepancies in any of the comparisons, the fieldworker had transcribed the data accurately. In this instance, the case required no further investigation, and was highlighted as ‘validated’. If a discrepancy was recognized in any of the comparisons, the SDV case was highlighted as a ‘query’. A query had to be resolved if the SDV case was to be validated. Some queries we were able to resolve immediately, and others required further investigation.

A query was also raised, if, upon examining a source document, the study protocol had not been complied with. This gave an indication of the success with which protocol was being undertaken throughout the trial, for example, legibility of digital images of source documents and health-worker completion rate of the CAPS sticker. Any areas of non-compliance were passed onto the clinical trial manager. The clinical trial manager highlighted these areas to fieldworkers to improve compliance.

The digital images of the pages of the health passport were examined for a finding of pneumonia. A positive finding of pneumonia was recorded if ‘pneumonia’, ‘lower respiratory tract infection’, or ‘acute lower respiratory infection’ was specified in the health passport, and related to the same episode recorded in the master database. If none of these terms were specified for the episode recorded in the master database or there was no digital image available since the last study visit, a negative finding of pneumonia was recorded. If a negative finding of pneumonia was recorded, the SDV case was highlighted as a query.

The digital image of the page of the health passport where the CAPS sticker had been inserted was examined for a finding of pneumonia. A positive finding was recorded if the information in the CAPS sticker related to the same episode recorded in the master database. If the information did not relate to the episode recorded in the master database or there was no digital image available of the CAPS sticker, a negative finding was recorded. Given that the CAPS sticker may or may not have been completed by the health worker, a negative finding in the CAPS sticker was highlighted as a query only if there was no digital image available.

In each SDV case, the severity classification and eight clinical indicators in the digital image of the CAPS sticker were compared with the data in the master database. If the data were consistent, a positive finding was recorded. If there was a discrepancy, a negative finding was recorded, and the SDV case was highlighted as a query.

## Results

There were 664 episodes of pneumonia recorded in the CAPS master database. Of the 664 episodes of pneumonia, 611 (92%) had a positive finding in the health passport and 53 (8%) had a negative finding in the health passport (one had no digital image available of the health passport) (Table 1). All 53 SDV cases that had a negative finding in the health passport were raised as queries.

**Table 1:** Occurrence of pneumonia, transcription of severity classification, and transcription of clinical indicators. The occurrence of episodes of pneumonia, transcription of severity classification, and transcription of clinical indicators in the source documents compared with the master database.

	Health passport	CAPS sticker
**Digital images available**	663 (100%)	530 (80%)
**Digital images unavailable**	1 (0%)	134 (20%)
Total cases	664	664
**Positive finding of pneumonia**	611 (92%)	280 (42%)
**Negative finding of pneumonia**	53 (8%)	384 (58%)
Total cases	664	664
**Correct transcription of severity classification**	N/A	237 (85%)
**Incorrect transcription of severity classification**	N/A	43 (15%)
Total cases	N/A	280
**Correct transcription of clinical indicators**	N/A	264 (94%)
**Incorrect transcription of clinical indicators**	N/A	16 (6%)
Total cases	N/A	280

Of the 664 episodes of pneumonia, 280 (42%) had a positive finding in the CAPS sticker and 384 (58%) had a negative finding in the CAPS sticker (134 had no digital image available of the CAPS sticker/ 250 had a digital image available) (Table 1). All 134 SDV cases that had no digital image available of the CAPS sticker were raised as queries. Of the 280 episodes of pneumonia recorded in the CAPS sticker, 43 had an incorrect severity classification transcribed in the master database (15%), 16 had at least one incorrect clinical indicator transcribed in the master database (6%). The 59 SDV cases with an incorrect transcription of the severity classification or clinical indicators were raised as queries.

There were 271 cases with a positive finding of pneumonia in both the health passport and the CAPS sticker (41%). There were 44 cases with a negative finding in both the health passport and the CAPS sticker (7%). There were 620 cases which had a positive finding in at least one source document (93%). The digital images of the pages of the health passport and CAPS stickers were clear and legible with few exceptions.

## Discussion

The use of a smartphone to capture data on the eCRF and digital images of the health passport and CAPS sticker allowed correction of transcription errors from the UK without the need for any additional visits. As the digital images of the source documents were clear and legible the data were easily verifiable.

The process of comparing data in the master database with data in the digital images of the source documents took approximately one to two minutes for each SDV case. Although this process was quick and simple, transferring the large volume of digital images, to be accessed in the UK, required many hours of work by the data managers. Because of slow connection speeds in Malawi, data were transferred on an ad-hoc basis. Furthermore, because ODK Collect stored images as a binary data type known as BLOB, these had to be extracted to convert them back into an image file. This manual process proved to be time-consuming. Data managers also developed the web-based SDV application, in which the data was viewed and the results of SDV recorded.

Eight percent of episodes of pneumonia recorded in the master database were not identified in the digital images of the health passport. It is likely that this is explained by missing digital images of the health passport, either not captured in the field or lost during processing. To achieve high quality documentation, we aim to investigate these instances with a new set of digital images at the next follow-up visit. Sixteen percent of SDV cases with a completed CAPS sticker had at least one incorrect value in severity classification or clinical indicators. In these instances, it was possible to amend the master database immediately, without further investigation. Although the SDV was limited to primary and secondary outcomes and study protocol monitoring, it could be used for a range of tasks to ensure the validity and integrity of the trial, including adverse event reporting, consent documentation, and regulatory compliance.

This methodology was successfully implemented in a challenging research setting. There had been some skepticism, given assumptions around the lack of infrastructure to support this technologically advanced approach. Where there had been some concern that fieldworkers recruited in rural settings of Malawi would lack experience with mobile technology, it was well accepted by all those who participated. This is in agreement with King et al. in a similar rural setting in Malawi, who found that fieldworkers preferred electronic data capture to traditional paper collection methods [19].

The CAPS sticker was included to help maximize the completeness and quality of information recorded for each episode of pneumonia (severity classification, symptoms and signs, and outcome). If we consider that completing the CAPS sticker was a voluntary additional exercise for health workers, who often work in busy health facilities, our finding that 42% of episodes of pneumonia recorded in the master database had a completed CAPS sticker in addition to the health passport, demonstrates this worked well. We provided pulse oximeters for the use in the assessment of pneumonia, locally tailored educational sessions, and a contribution to the medical equipment fund for the district hospitals involved.

## Conclusion

The use of an eCRF and digital images of the source documents allowed SDV to be conducted remotely without the need for additional field visits. Although no formal analysis of costs was made, it is likely that the use of widely available low-cost mobiles devices was more efficient than labour- intensive traditional paper-collection methods. These methods will become more important as electronic data capture becomes the norm, particularly in challenging research settings. We recommend these approaches in similar settings, especially those with health endpoints.

## Supporting Information

S1 Supporting Information The results of Source Data Verification (SDV). A complete table of the results of SDV for the present work.
(XLSX)
